# Ankle fracture classification using deep learning: automating detailed AO Foundation/Orthopedic Trauma Association (AO/OTA) 2018 malleolar fracture identification reaches a high degree of correct classification

**DOI:** 10.1080/17453674.2020.1837420

**Published:** 2020-10-26

**Authors:** Jakub Olczak, Filip Emilson, Ali Razavian, Tone Antonsson, Andreas Stark, Max Gordon

**Affiliations:** Karolinska Institute, Institution for Clinical Sciences, Danderyd University Hospital, Stockholm, Sweden

## Abstract

Background and purpose — Classification of ankle fractures is crucial for guiding treatment but advanced classifications such as the AO Foundation/Orthopedic Trauma Association (AO/OTA) are often too complex for human observers to learn and use. We have therefore investigated whether an automated algorithm that uses deep learning can learn to classify radiographs according to the new AO/OTA 2018 standards.

Method — We trained a neural network based on the ResNet architecture on 4,941 radiographic ankle examinations. All images were classified according to the AO/OTA 2018 classification. A senior orthopedic surgeon (MG) then re-evaluated all images with fractures. We evaluated the network against a test set of 400 patients reviewed by 2 expert observers (MG, AS) independently.

Results — In the training dataset, about half of the examinations contained fractures. The majority of the fractures were malleolar, of which the type B injuries represented almost 60% of the cases. Average area under the area under the receiver operating characteristic curve (AUC) was 0.90 (95% CI 0.82–0.94) for correctly classifying AO/OTA class where the most common major fractures, the malleolar type B fractures, reached an AUC of 0.93 (CI 0.90–0.95). The poorest performing type was malleolar A fractures, which included avulsions of the fibular tip.

Interpretation — We found that a neural network could attain the required performance to aid with a detailed ankle fracture classification. This approach could be scaled up to other body parts. As the type of fracture is an important part of orthopedic decision-making, this is an important step toward computer-assisted decision-making.

Ankle fractures are recognized among the most common fractures, with peak incidence between 15 and 29 years (67 per 100,000 person-years) and elderly women ≥ 60 years (174 per 100,000 person-years) (Westerman and Porter 2007, Thur et al. 2012). Efforts to classify ankle fractures in clinically relevant entities have a long history, ending in 3 classic systems, i.e., the Lauge-Hansen (Hansen 1942), Danis–Weber, and the AO/OTA classifications (Association Committee for Coding and Classification 1996; Budny and Young 2008), where the Danis–Weber with its A, B, and C classes is probably the most used in everyday practice.

The most recent update for the AO/OTA classification system was published in 2018 (Meinberg et al. 2018). The AO/OTA system contains classifications for the entire body. The ankle is divided into (1) malleolar, (2) distal tibia, and (3) fibular fractures. For malleolar fractures, the subcategories correspond to the Danis–Weber ABC classification (Hughes et al. 1979) with the addition of a suffix of 2 digits (range 1–3), e.g., the common intra-syndesmotic B-injury without widening of the mortise corresponds to the B1.1 class. The numbers correspond roughly to the severity of each fracture.

The complexity of this classification makes it difficult to learn and apply, limiting inter-observer reliability and reproducibility (Fonseca et al. 2017). This has hindered its use in an everyday clinical setting, suggesting the need for better aid during the classification.

During recent years, the resurgence of neural networks, a form of artificial intelligence (AI), has proven highly successful for image classification. In some medical image classification applications neural networks attain (Olczak et al. 2017, Kim and MacKinnon 2018, Gan et al. 2019), and surpass, human expert performance (Esteva et al. 2017, Lee et al. 2017, Chung et al. 2018, Urakawa et al. 2019). Machine learning and neural networks are also becoming more commonplace research tools in orthopedics. They hold great potential, as the diagnostic underpinning and intervention decision relies heavily on medical imaging (Cabitza et al. 2018). The strength of these learning algorithms is their ability to review a vast number of examinations and examples, and the speed and consistency with which they can review each examination and at the same time remember thousands of categories without issue.

We therefore hypothesized that a neural network can learn to classify ankle fractures according to the AO/OTA 2018 classification from radiographs.

## Method

### Study design

**Figure 1.**
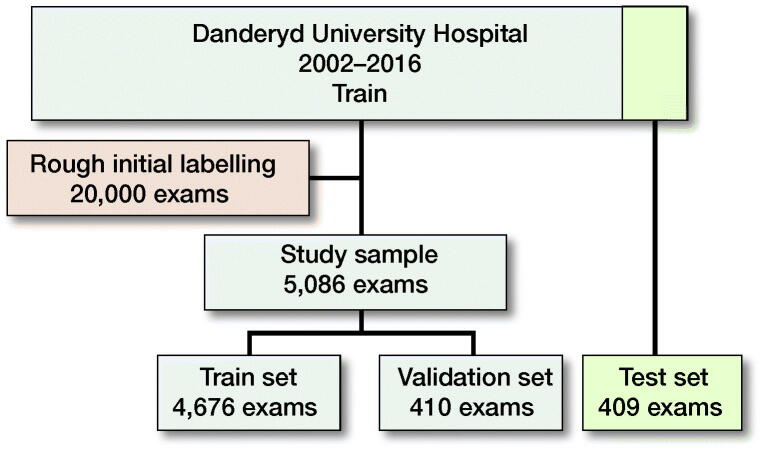
Data flowchart.

The initial dataset consisted of deidentified orthopedic radiographic examinations of various anatomical regions taken between 2002 and 2016 at Danderyd University Hospital in Stockholm, Sweden. Through using the radiologist’s report, we identified images with a high likelihood of fracture, comminution, dislocation, and/or displacement. Based on these categories, we randomly selected a study set of 5,495 ankle examinations where the categories allowed for selecting cases with a higher likelihood of pathology. We introduced this bias to include as many sub-classifications of fractures in the dataset as possible. From the study set, we selected 400 random patients (411 examinations) to include into the test set. 75% were chosen for having reports suggesting a fracture. Similarly, we chose the training and validation sets to have approximately 50% chance of having a fracture (Figure 1). This introduced a selection bias towards pathology, as the primary task was to distinguish different types of fractures and not just the presence of a fracture.

We excluded any examination within 90 days of a previously included examination, to ensure that the same fracture was not included more than once, e.g., pre/post reposition/surgery. We further excluded the few pediatric fractures (defined as open physes) as nearly all patients at the hospital are older than 15 years. 145 examinations were excluded from training and 2 examinations from testing. The final study set included 4,676 examinations in the training set and 409 examinations in the test set.

### Labeling and outputs

All examinations selected for the study set were manually reviewed and labelled according to the AO/OTA classification (Meinberg et al. 2018) down to subgroup but excluding subgroup qualifiers. We use the term class for a possible classification outcome, as a summary term for bone, segment, type, group, and subgroup outcome, and specify more clearly when necessary. This means we have 39 classes of malleolar fractures with 3 types (A–C), 3 groups per type (1–3) for each class, and 27 subgroups (3 subgroups per group).

Each exam in the training set was reviewed by a minimum of 2 out of 5 reviewers (FE, AS, MG, JO, TA) using a custom-built image-labeling platform displaying the entire full-scale examination together with the original radiologist report. Reviewer FE was a 5th-year medical student, JO and TA were medical doctors. FE, TA, and JO were specifically trained for the task of labeling radiographic ankle examinations according to the AO/OTA 2018 classification for ankle fractures and labeled between 2,000 and 4,000 examinations each. MG is a senior orthopedic surgeon specializing in orthopedic trauma and AS is a senior orthopedic surgeon. In a second step, all examinations classified as having fractures were rereviewed by MG before being added to the training set. The test set was reviewed by MG and AS. We required a minimum of at least 5 fractures per outcome in the training dataset before including that outcome.

The AO classification is partially ligamentous based and as ligaments are not visible on radiographs we therefore used proxies for these classes. For infra-syndesmotic lateral malleolar fractures, if the avulsion fragment was ≤ 3 mm from the tip we classified it as A1.1, 3–10 mm from the tip as A1.2, and ≥ 10 mm as A1.3. As the B1.1 and B1.2 class differ only by syndesmotic injury, information that was not available to us, we chose to separate these by the presence of a step-off in the fracture that could suggest a rotation of the distal fragment. Another important note is that we defined B2.1 based on the presence of a widening of the ankle fork, and this can thus be falsely negative if the ankle has been well repositioned in a cast.

Visible fractures of the tibia and fibula were classified as far as possible. Only the complete ankle examinations were included, but no additional examinations of the tibia, fibula, or the foot.

In the AO/OTA 2018 version there is an inherent overlap between fibular fractures of the distal end segment (4F3) and fractures of the lateral malleolus (44A–C). A distal end segment fibular fracture (4F3) cannot necessarily be distinguished from ankle fractures involving the distal fibula (44A–C). If the fracture was deemed not to be associated with an ankle fracture it was coded as a fibular fracture (4F) and if it was deemed to be part of an ankle fracture it was coded as (44A–C), as by Meinberg et al. (2018). The final verdict was decided by MG.

The 2018 AO/OTA revision has separate classifications for epiphyseal, metaphyseal, and diaphyseal fractures, and it was possible to have multiple labels when multiple fractures and fracture systems were present.

### Data set

The training data consisted of labeled examinations passed to the network. A subset of the initial dataset was randomly selected for the test set and was never used during training or validation. We used a biased selection, 75% of fractures, to increase the likelihood of selecting rare fracture types. The test set was manually and independently classified and verified by MG and AS using the same platform as in the training set. Any cases where there was disagreement were then subsequently re-reviewed for a consensus on the final classification of the test set (Table 1).

**Table 1.** Base distribution of fractures according to the AO classification. Values are count (%)

	Train (n = 4,941)			Test (n = 409)		
Fracture type	Yes	Maybe	No	Yes	Maybe	No
Fracture	2,156 (44)	121 (2)	2,664 (54)	306 (75)	13 (3)	90 (22)
Malleolar (44)	1,696 (34)	63 (1)	3,182 (64)	210 (51)	6 (1)	193 (47)
Tibia distal (43)	254 (5)	6 (0)	4,681 (95)	63 (15)	2 (0)	344 (84)
Fibula (4F2–3)	129 (3)	3 (0)	4,809 (97)	37 (9)	0 (0)	372 (91)
Tibia diaphyseal (42)	88 (2)	0 (0)	4,853 (98)	27 (7)	0 (0)	382 (93)
Other bone	210 (4)	47 (1)	4,684 (95)	35 (9)	5 (1)	369 (90)

“Other bone” generally indicates a visible fracture of the foot. It was possible for an examination to have multiple fracture labels.

### Validation set and active learning

Before each round of training a new validation set of 400 patients was randomly selected. Based on the validation outcome we:

- re-validated categories for training images where the network performed poorly to ensure the quality of training labels;
- used targeted sampling via the network outputs combined with specific searches in the radiologist’s reports to extend the original training dataset for low-performing categories;
- implemented active learning, where categories with low performance despite having plenty of training examples were targeted with more data and targeted review of training labels during training.

### Image input

The labeled radiographic images were scaled down with retained proportions, so that the largest side had 256 pixels. If the image was not square, the shorter side was extended with black pixels resulting in a 256 · 256 square proportionally scaled copy.

### Neural network design

We used a modified ResNet architecture (He et al. 2015) with a layered structure, which was randomly initiated at the beginning of the experiment. The network, training setup including overfitting strategies, is presented in Table 2 (Supplementary data). Each output had its own 2-layer subnetwork and a margin loss. To merge outcomes from various images within the same examination we used the max. function, i.e., if the network predicted 2 or more outcomes, the one with the highest predicted likelihood was selected, ensuring each examination had a unique outcome. The “maybe” outcome was included in the margin loss during training, but was categorized as “no fracture” during validation and testing. Each outcome was calculated separately so classifying a fracture as type B did not follow from classifying a fracture as group B1, which in turn was a separate classification from subgroup B1.1. However unlikely, it is possible for the network to classify a fracture as a type B fracture (between types A and C) and at the same time determine that it is a C1.1 fracture for subgroup classification.

**Table 3.** Distribution of malleolar fractures by type (44A–C), specified by type, group, and subgroup. Values are count (%) for samples > 100

AO type	Train (n = 4,941)	Test (n = 409)
44A (483 train and 31 test cases)		
1.1	78 (22)	6
1.2	165 (46)	7
1.3	114 (32)	9
2.1	105 (93)	5
2.2	1 (1)	–
2.3	7 (6)	2
3.1	11	–
3.3	2	2
44B (1,015 train and 136 test cases)		
1.1	385 (74)	39
1.2	132 (25)	26
1.3	6 (1.1)	2
2.1	99 (44)	20
2.2	105 (47)	16
2.3	19 (8.5)	2
3.1	76 (28)	12
3.2	152 (56)	13
3.3	41 (15)	6
44C (255 train and 47 test cases)		
C1		
1.1	85 (67)	17
1.2	20 (16)	5
1.3	22 (17)	2
2.1	30	6
2.2	21	3
2.3	39	9
3.1	10	3
3.2	9	1
3.3	19	1

### Outcome performance/statistics

The primary outcome was receiver-operating curve (ROC) area under curve (AUC) accuracy for AO/OTA malleolar fracture type, group, and subgroup or no fracture outcome for the complete examination. Secondary outcomes were fibular and tibial AO/OTA classes, as well as any foot fracture when present. These were secondary outcomes as we did not look at the complete examinations, e.g., proximal femur or foot examinations. To test the diagnostic accuracy of the neural network, we also calculated the sensitivity, specificity, and Youden’s index (Youden 1950) for each outcome. There is no consensus as to what an adequate J is, but bigger J is generally more useful. Chung et al. (2018) found that J > 0.71 indicated performance superior to an orthopedic surgeon for detecting any fracture in hip radiographs. Two-way interobserver reliability Cohen’s kappa and percentage agreement was computed between all observers. The overall best performing model (highest AUC) on the validation set was used for final testing on the test set. As there is a large number of categories we also present a weighted mean for groups. The weighting is according to the number of cases as we want small categories that may perform well by chance to have less influence on the weighted mean; for AUC the calculation was: 
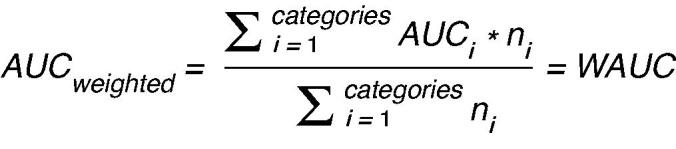


Only outcomes with ≥ 2 cases in the test set were evaluated during testing. Main outcomes were classes A–C, group A1–C3, subgroup A1.1–C3.3.

### Ethics, funding, and potential conflicts of interest

This study was approved by the Regional Ethics Committee fort Stockholm, Sweden (Dnr. 2014/453-31/3, April 9, 2014). This project was supported by grants provided by Region Stockholm (ALF project), the Swedish Society of Doctors (Svenska Läkaresällskapet) and by the Karolinska Institute. AS and MG are co-founders and shareholders in DeepMed AB. AR is a shareholder in DeepMed AB.

## Results

5,495 radiographic examinations were used in the experiment. 5,086 examinations were used for training and validation and 409 examinations (400 unique patients) were withheld in the test set, with no patient overlap.

**Figure 2.**
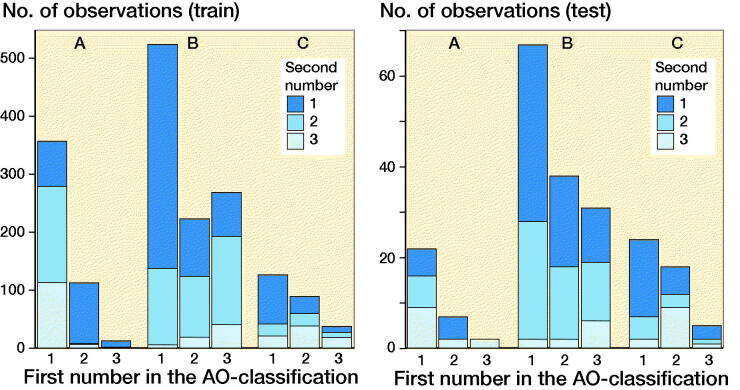
Distribution of AO classes in the malleolar fracture data.

In the combined data, there were 2,462 examinations with a fracture. Malleolar fractures were by far the most prevalent fractures (1,906 out of 2,462 fractures) and the majority of them were type B injuries (1,147), followed by type A injuries (456) and type C injuries (300). The training set had 1,753 malleolar fractures for 39 possible outcomes, averaging 48 positive training cases per outcome, though some classes had more fractures than others (Table 3 and Figures 1 and 2).

### Main results

32 out of 39 outcomes had 2 or more examinations in the test set. Most outcomes were possible to train and most classes that disappeared had too few test cases (Table 4).

**Table 4.** Outcome measures for the most important groups and weighted average AUC for each malleolar AO type, group, and subgroup combined

	Cases	Sensitivity	Specificity		
AO type	n = 409	(%)	(%)	Youden’s J	AUC (95% CI)
44A					
Base	32	73	81	0.54	0.81 (0.72–0.88)
1	22	88	75	0.63	0.87 (0.77–0.94)
1.1	6	75	93	0.68	0.87 (0.70–0.98)
1.2	7	80	83	0.63	0.79 (0.54–0.94)
1.3	9	75	88	0.63	0.84 (0.70–0.95)
2	7	100	74	0.74	0.91 (0.83–0.97)
2.1	5	100	74	0.74	0.89 (0.80–0.97)
3	2	100	86	0.86	0.90 (0.83–0.96)
44B					
Base	137	89	88	0.77	0.93 (0.90–0.95)
1	67	90	88	0.77	0.93 (0.88–0.96)
1.1	39	87	84	0.71	0.89 (0.85–0.93)
1.2	26	92	85	0.77	0.90 (0.81–0.96)
2	38	82	84	0.65	0.87 (0.80–0.92)
2.1	20	100	72	0.72	0.87 (0.83–0.92)
2.2	16	88	74	0.62	0.82 (0.68–0.91)
2.3	2	100	98	0.98	0.99 (0.97–1.00)
3	32	78	90	0.68	0.90 (0.85–0.94)
3.1	12	83	75	0.58	0.79 (0.63–0.90)
3.2	13	92	82	0.74	0.91 (0.84–0.96)
3.3	6	100	91	0.91	0.96 (0.93–0.98)
44C					
Base	47	74	90	0.65	0.86 (0.79–0.92)
1	24	75	79	0.54	0.83 (0.72–0.91)
1.1	17	76	85	0.61	0.86 (0.74–0.94)
1.2	5	80	92	0.72	0.89 (0.77–0.97)
1.3	2	100	88	0.88	0.92 (0.86–0.97)
2	18	100	72	0.72	0.91 (0.86–0.95)
2.1	6	83	93	0.76	0.91 (0.79–0.98)
2.2	3	100	88	0.88	0.96 (0.88–1.00)
2.3	9	100	77	0.77	0.88 (0.84–0.92)
3	5	100	88	0.88	0.95 (0.90–0.98)
Malleolar	216	86	90	0.76	0.92 (0.89–0.95)
Weighted mean AUC					
A					0.84
B					0.90
C					0.87
Malleolar					0.90

Criterion based on Youden’s Index (Youden 1950, Aoki et al. 1997, Shapiro 1999, Greiner et al. 2000) defined as

$$YI\left(c\right)=max_{c}\left(Se\left(c\right)+Sp\left(c\right)–1\right).$$

This is identical (from an optimization point of view) to the method that maximizes the sum of sensitivity and specificity (Albert 1987, Zweig and Campbell 1993) and to the criterion that maximizes concordance, which is a monotone function of the AUC.

For malleolar fractures, weighted mean AUC came to 0.90 with varying 95% confidence intervals (CI) for individual classes. The network could identify malleolar fractures with an AUC 92 (CI 0.89–0.95). For malleolar fracture overall best performance was achieved for type B (1,015 in the training and 136 in the test set) injuries with AUC 0.93 (CI 0.90–0.95), then type C (255 cases in the training and 47 cases in the test set) with AUC 0.86 (CI 0.78–0.92), and then type A (483 in the training and 31 in the test set) with AUC 0.81 (CI 0.72–0.88).

Type A injuries exhibited the poorest results with weighted average AUC 0.84. It was not possible to evaluate subgroups A2.1, A2.3, and subgroups of A3. Average AUC for type B injuries was 0.90 and all classes, except the subgroup B13, were evaluated. Weighted average AUC for type C injuries was 0.87 but it was not possible to evaluate subgroups to C3. Despite there being almost twice as many type A fractures in the data set there were fewer type A fractures in the test set, which resulted in few outcomes for type A fractures.

### Other anatomies

The number of fractures in the other anatomies did not allow for a detailed analysis for many of the classes. In the test set the second most common fracture group was the distal tibia group with weighted average AUC 0.90. We found similar values for the isolated fibular and tibial diaphysis fractures. The foot fractures were somewhat less performant, mostly due to metatarsal fractures (see Supplementary data).

### Other analyses

**Figure 3.**
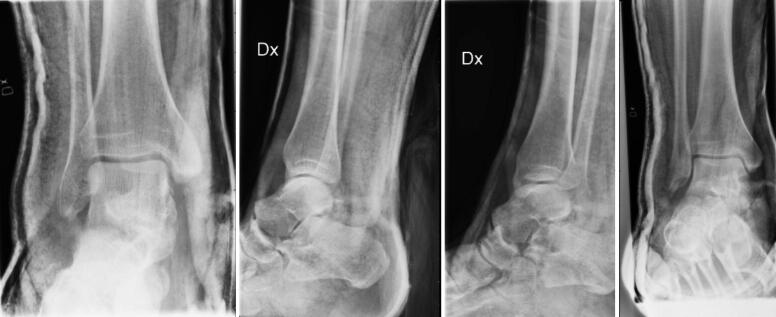
The network failed to identify this image as a malleolar type A fracture. Among the malleolar fractures it was predicted as a type C fracture.

**Figure 4.**
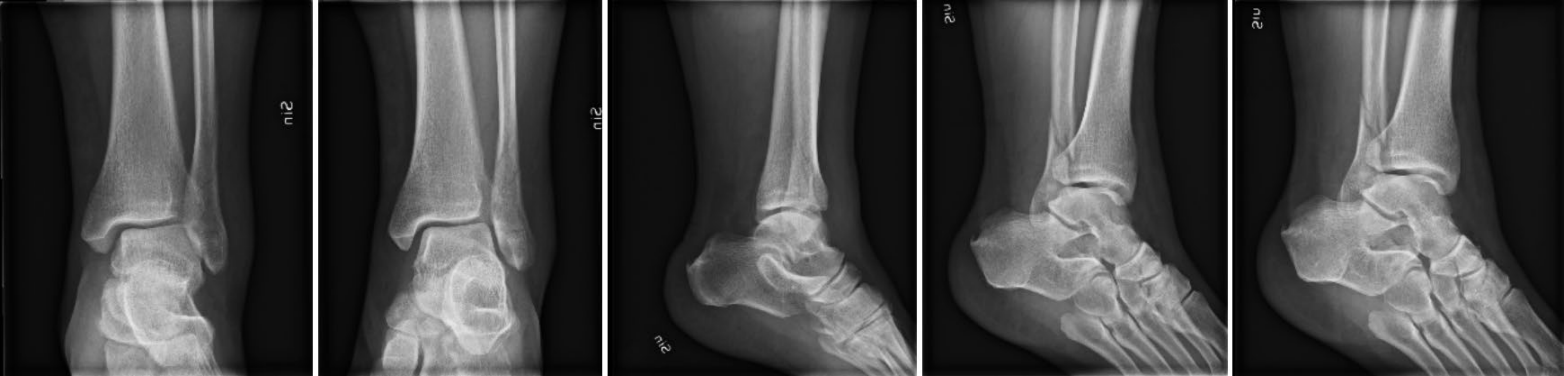
The examination should have been a malleolar type C fracture but the network predicted type B fracture.

Overall Cohen’s kappa between reviewers was 0.65 (and 0.55 on the AO classification task) (see Supplementary data). When reviewing the failed images there was no obvious pattern. The presence of casts was common (Figure 3) or discrete findings (Figure 4) were common but we could not see any clear pattern that the failures followed.

## Discussion

This study is the first, to our knowledge, that classifies fractures according to the AO/OTA classification, and ankle fractures in particular, using machine learning. We believe that an average AUC 0.90 for the relatively complex AO/OTA classification task, on a small training set with many categories, is a good outcome.

This study shows the potential benefits of an AI classification, where complex classifications can become commonplace to the benefit of patients and their treatment. We have shown that a neural network, using a combinatory approach with different machine learning methods and targeted labeling, can learn even rare fracture types.

In the AO/OTA classification, C injuries tend to be more complex and severe than type B injuries, which in turn are worse than type A injuries. Higher group and subgroup numbers also tend to entail more severe and complex injuries. We found that malleolar type A injuries decreased in frequency with severity, and that there were fewer type A than type B injuries. One reason for this was that many minor fractures, e.g., simple avulsion fragments, are a form of distortion that, despite being commonplace, are difficult to diagnose through radiographs.

Fonseca et al. (2017) found a kappa of 0.38 for the AO classification (not subgroups) whereas our study found kappa 0.55 between the human reviewers MG and AS. In a separate test, 388 of the examinations in the test set were reviewed by a resident emergency medical specialist (TA). Kappa for this subset of the training set was 0.53 or an agreement of 92%. One reason for this could be that reviewers usually had access to the radiologist’s report, probably improving kappa, while the network never did. While the report never specified AO classification and mostly helped identify discrete fractures, it also helped fill in the lack of additional patient information. In addition, the very unsymmetrical distribution of outcomes (marginal probabilities for each individual class) between AO classes (e.g., C3.3 is much less uncommon than B1.1) likely unduly penalizes kappa for the AO classification task (Delgado and Tibau 2019). Compared with Juto et al. (2018) we found a percentile agreement of ≥ 91% for all levels (fracture type, group, and subgroup) between observers. Both Fonseca et al. (2017) and Juto et al. (2018) used the previous AO/OTA classification.

Detecting a fracture was easy for humans and computer alike and there was great agreement, but in line with other studies AO/OTA classification is complicated as is shown by the declining kappa. This strengthens the case for an automated classification system that can assist in making uniform classifications. Overall, the network was good at classifying ankle fractures and its subgroups though some subclasses were difficult and many had insufficient data.

### Limitations

We relied on only radiographs and the radiologist’s report, which does not fully allow for discrimination between the AO/OTA classifications, especially where ligamentous injuries are important. However, as most ankle fractures will never undergo a CT or MRI examination, extracting additional chart information would have little impact on the outcome. CT and MRI are also not performed randomly on fractures and including them in our results, when available, would introduce an information bias. We also strongly believe that clinicians will always have to add their clinical exam to the interpretation even with these new technologies, as some information simply is not present in a radiographic image.

This study reports the outcome of the top classification, the highest AUC. For many malleolar subgroups the difference is small and it would make sense to present additional likely outcomes, in particular outcomes where the differences are only in ligamentous injuries, alongside each other—for example B1.1 and B1.2. A repositioned or stabilized fracture can hide a previously obvious ligamentous injury, changing the classification.

Our data entailed a selection bias towards pathological material and did not represent the average population. Despite this, there were insufficient cases for many subclasses and for some outcomes the statistical significance and confidence intervals were difficult to assess. Uncommon pathologies are problematic for any human observer or deep learning system. We have combated this by selecting new cases for annotation where the network has either (1) difficulties distinguishing a category, or (2) high likelihood of a rare fracture class, a form of active learning. This interactive approach to machine learning proved useful and could be repeated, and adding more data could help target rare fractures.

The human observers had access to full-scale radiographs and reports whereas the network, at best, had proportionally scaled 256 · 256 representations. Despite this limitation, many of the categories were correctly identified. We believe that this is most likely due to the fact that the network reviews each image and thus is able to find even tiny changes. We chose this approach as our experience has indicated that increasing image size has little benefit. Similarly, we have tried some different permutations of the network structure with mostly similar outcomes. It is important to keep in mind that the literature surrounding deep learning is vast and there are many interesting network designs that could be tested. Regardless, we believe that the chosen structure fulfills our aim, to find a network that can help clinicians to use complex fracture classifications on an everyday basis.

Despite having a large dataset and actively searching for pathology we found it hard to find an adequate number of fractures for many of the classes. While we can retrain the network to fit new categories, it is important to remember that fractures in case reports and other rare entities will be a challenge for deep learning applications and clinicians alike.

### Generalizability

The source population was dominated by a Caucasian population. We excluded only examinations with open physes and believe that our results generalize well in a regular clinical setting, though we would expect more negative cases and simple fractures than in our material. The clinical performance of the algorithm may therefore differ from the sample performance. Our results also extend to the Danis–Weber classification to the extent that it is a subset of the AO classification.

### Interpretation

A neural network can learn the AO/OTA classification from relatively few training examples. Even with this small data set we find that we can achieve high predictive accuracy for most categories. The strength of an AI model is the ability to further improve the model by adding more training cases and its potential for uniform classification.

## Supplementary Material

Supplemental Material

## Supplementary data

Table 2, inter-rater reliability (IRR) results, and data on other fracture classes are available as supplementary data in the online version of this article, http://dx.doi.org/10.1080/17453674. 2020.1837420

## References

[CIT0001] Albert . Multivariate interpretation of clinical laboratory data. Boca Raton, FL: CRC Press; 1987.

[CIT0002] Aoki K , Misumi J , Kimura T , Zhao W , Xie T . Evaluation of cutoff levels for screening of gastric cancer using serum pepsinogens and distributions of levels of serum pepsinogen I, II and of PG I/PG II ratios in a gastric cancer case-control study. J Epidemiol 1997; 7(3): 143–51. 9337512 10.2188/jea.7.143

[CIT0003] Association Committee for Coding and Classification. Fracture and dislocation compendium . Orthopaedic Trauma Association Committee for Coding and Classification. J Orthop Trauma 1996; 10(Suppl. 1): v-ix, 1–154. 8814583

[CIT0004] Budny A M , Young B A . Analysis of radiographic classifications for rotational ankle fractures. Clin Podiatr Med Surg 2008; 25(2): 139–52. 18346587 10.1016/j.cpm.2007.11.003

[CIT0005] Cabitza F , Locoro A , Banfi G . Machine learning in orthopedics: a literature review. Front Bioeng Biotechnol [Internet] 2018 Jun 27 [cited 2019 Oct 29]; 6. Available from: https://www.ncbi.nlm.nih.gov/pmc/articles/PMC6030383/ 10.3389/fbioe.2018.00075 PMC6030383 29998104

[CIT0006] Chung S W , Han S S , Lee J W , Oh K-S , Kim N R , Yoon J P , et al. Automated detection and classification of the proximal humerus fracture by using deep learning algorithm. Acta Orthop 2018; 89(4): 468–73. 29577791 10.1080/17453674.2018.1453714 PMC6066766

[CIT0007] Delgado R , Tibau X-A . Why Cohen’s Kappa should be avoided as performance measure in classification. PLOS ONE 2019; 14(9): e0222916. 31557204 10.1371/journal.pone.0222916 PMC6762152

[CIT0008] Esteva A , Kuprel B , Novoa R A , Ko J , Swetter S M , Blau H M , et al. Dermatologist-level classification of skin cancer with deep neural networks. Nature 2017; 542(7639): 115. 28117445 10.1038/nature21056 PMC8382232

[CIT0009] Fonseca L , Nunes I , Nogueira R , Martins G , Mesencio A , Kobata S . Reproducibility of the Lauge-Hansen, Danis–Weber, and AO classifications for ankle fractures. Rev Bras Ortop Engl Ed 2017; Dec 1: 53. 10.1016/j.rboe.2017.11.013 PMC5771788 29367914

[CIT0010] Gan K , Xu D , Lin Y , Shen Y , Zhang T , Hu K , et al. Artificial intelligence detection of distal radius fractures: a comparison between the convolutional neural network and professional assessments. Acta Orthop 2019; 90(4): 394–400. 30942136 10.1080/17453674.2019.1600125 PMC6718190

[CIT0011] Greiner M , Pfeiffer D , Smith R D . Principles and practical application of the receiver-operating characteristic analysis for diagnostic tests. Prev Vet Med 2000; 45(1): 23–41. 10802332 10.1016/s0167-5877(00)00115-x

[CIT0012] Guillaumin M , Verbeek J , Schmid C . Multimodal semi-supervised learning for image classification. In: 2010 IEEE Computer Society Conference on Computer Vision and Pattern Recognition [Internet]. San Francisco, CA: IEEE; 2010 [cited 2020 Jan 14]. p. 902–9. Available from: http://ieeexplore.ieee.org/document/5540120/

[CIT0013] Hansen N L . Ankelbrud i genetisk Diagnose og Reposition: experimental-chirurgiske og radiografiske Undersøgelser Repositionsforsøg i Kliniken. Copenhagen: E. Munksgaard; 1942.

[CIT0014] He K , Zhang X , Ren S , Sun J . Delving deep into rectifiers: surpassing human-level performance on ImageNet Classification. 2015 [cited 2016 Aug 19]. p. 1026–34. Available from: http://www.cv-foundation.org/openaccess/content_iccv_2015/html/He_Delving_Deep_into_ICCV_2015_paper.html

[CIT0015] Hughes J L , Weber H , Willenegger H , Kuner E H . Evaluation of ankle fractures: non-operative and operative treatment. Clin Orthop Relat Res 1979; (138): 111. 445892

[CIT0016] Izmailov P , Podoprikhin D , Garipov T , Vetrov D , Wilson A G . Averaging weights leads to wider optima and better generalization. ArXiv180305407 Cs Stat [Internet] 2019 Feb 25 [cited 2019 Nov 16]. Available from: http://arxiv.org/abs/1803.05407

[CIT0017] Juto H , Nilsson H , Morberg P . Epidemiology of adult ankle fractures: 1756 cases identified in Norrbotten County during 2009–2013 and classified according to AO/OTA. BMC Musculoskelet Disord 2018; 19(1): 441. 30545314 10.1186/s12891-018-2326-x PMC6293653

[CIT0018] Kim D H , MacKinnon T . Artificial intelligence in fracture detection: transfer learning from deep convolutional neural networks. Clin Radiol 2018; 73(5): 439–45. 29269036 10.1016/j.crad.2017.11.015

[CIT0019] Lee J-G , Jun S , Cho Y-W , Lee H , Kim GB , Seo JB , et al. Deep Learning in Medical Imaging: General Overview. Korean J Radiol 2017; 18(4): 570–84. 28670152 10.3348/kjr.2017.18.4.570 PMC5447633

[CIT0020] Meinberg E G , Agel J , Roberts C S , Karam M D , Kellam J F . Fracture and dislocation classification compendium—2018. J Orthop Trauma 2018; 32(Suppl. 1): S1–S170. 10.1097/BOT.0000000000001063 29256945

[CIT0021] Olczak J , Fahlberg N , Maki A , Razavian A S , Jilert A , Stark A , et al. Artificial intelligence for analyzing orthopedic trauma radiographs. Acta Orthop 2017; 88(6): 581–6. 28681679 10.1080/17453674.2017.1344459 PMC5694800

[CIT0022] Shapiro D E . The interpretation of diagnostic tests. Stat Methods Med Res 1999; 8(2): 113–34. 10501649 10.1177/096228029900800203

[CIT0023] Thur C K , Edgren G , Jansson K-Å , Wretenberg P . Epidemiology of adult ankle fractures in Sweden between 1987 and 2004. Acta Orthop 2012; 83(3): 276–81. 22401675 10.3109/17453674.2012.672091 PMC3369155

[CIT0024] Urakawa T , Tanaka Y , Goto S , Matsuzawa H , Watanabe K , Endo N . Detecting intertrochanteric hip fractures with orthopedist-level accuracy using a deep convolutional neural network. Skeletal Radiol 2019; 48(2): 239–44. 29955910 10.1007/s00256-018-3016-3

[CIT0025] Westerman R W , Porter K . Ankle fractures in adults: an overview. Trauma 2007; 9(4): 267–72.

[CIT0026] Youden W J . Index for rating diagnostic tests. Cancer 1950; 3(1): 32–5. 15405679 10.1002/1097-0142(1950)3:1<32::aid-cncr2820030106>3.0.co;2-3

[CIT0027] Zweig M H , Campbell G . Receiver-operating characteristic (ROC) plots: a fundamental evaluation tool in clinic medicine. Clin Chem 1993; 39(4): 561–77. 8472349

