# Supplementary material for: Ankle fracture classification using deep learning: automating detailed AO Foundation/Orthopedic Trauma Association (AO/OTA) 2018 malleolar fracture identification reaches a high degree of correct classification
Source: Acta Orthop. 2020 Oct 26;92(1):102–8. doi: 10.1080/17453674.2020.1837420 (PMC7919919; doi:10.1080/17453674.2020.1837420)
Supplement: Supplemental Material [file IORT_A_1837420_SM7117.pdf]

Supplementary data

Table 2a. Network

Layer type	Blocks	Kernel size	Filters	Group
ResNet block	1x2	5x5	32	Image
ResNet block	1x2	3x3	64	Image
ResNet block	4x2	3x3	64	Core
ResNet block	2x2	3x3	128	Core
ResNet block	2x2	3x3	256	Core
ResNet block	2x2	3x3	512	Core
Image max	1	–	–	Pool
Convolutional	1	1x1	72	Classification
Fully connected	1	–	4	Classification
Fully connected	1	–	4	Classification

Table 2b. Training set-up

Session	Epochs	Internal learning rate	Noise (%)	Teacher–student pseudolabels
Initalization	70	0.025	None	No
Noise	80	0.025	5	No
Teacher-student	40	0.005	5	Yes
Regularization	20	0.025	10	No
SWA	20x5	0.01	5	No

Table 2c. Overfitting strategy

Overfitting strategy	Description
Image jittering	Each image was rotated, flipped and randomly cropped during training
Random noise	We employed denoising autoencoder to regularize the visual representation manifold. The decoder and encoder have identical parameters and layers
Teacher–student network using alternate data	A form of semi-supervised training. A coexisting teacher network was allowed to learn the labels from both the image and the report. This allowed us to use the teacher's labels when images were not labeled. The teacher label's loss was reduced by 10% to avoid flooding the network with bad labels, as these were less certain than the manually labeled images. During the teacher–student session the data set was augmented unlabeled exams using a ratio of 1:2. During all sessions we switched between the ankle dataset and a similarly labeled dataset with wrist images that consisted of 17,511 exams. These were also augmented with unlabeled images with the same proportion between unlabeled and labeled in the ankle dataset (Guillaumin et al. 2010)
Stochastic weight averaging (SWA)	Cosine function for decreasing the learning rate, which was reset between each section of training. Once the learning rate leveled off, we trained for 5 series using stochastic weight averaging (Izmailov et al. 2019)
Active learning	Categories that performed poorly during training were actively reviewed during training to ensure accuracy and more examinations were added to improve those categories. Highest entropy over predictions was used as the sampling strategy for active learning

We used an untrained ResNet structure consisting of 26 layers. Batch normalization was applied to each convolutional layer and adaptive max. pool. The learning rate followed the cosine function.

 Acta Orthopaedica 2020; 91 (DOI: 10.1080/17453674.2020.1837420)

Inter-rater reliability (IRR) results

IRR between MG and AS for 409 observations. Average Cohen's kappa 0.65

	Cohen's kappa	% identifiedin the category Reviewer 1	Reviewer 2	Agreement
General				
Fracture	1.00	78	78	100
Malleolar	0.91	53	51	95
Tibia diaphyseal	0.57	7	3	96
Tibia distal	0.91	15	14	98
Fibula	0.84	9	9	98
Other bone	0.81	10	7	97
Malleolar				
A	0.63	9	5	95
B	0.82	33	35	92
C	0.73	10	11	95
A1	0.70	6	4	97
B1	0.63	17	11	91
B1.1	0.48	11	7	91
B1.2	0.22	6	4	93
B1.1 or B1.2	0.63	16	11	91
B3	0.51	9	6	93
C1	0.46	6	5	94
C2	0.55	3	5	96
C3	0.28	1	1	99

IRR between MG and TA for 388 observations. Average Cohen's kappa 0.60

	Cohen's kappa	% identifiedin the category Reviewer 1	Reviewer 2	Agreement
General				
Fracture	1.00	74	74	100
Malleolar	0.64	44	60	82
Tibia diaphyseal	0.60	4	2	98
Tibia distal	0.88	8	8	98
Fibula	0.56	4	3	97
Other bone	0.72	7	8	96
Malleolar				
A	0.48	8	18	88
B	0.72	28	39	87
C	0.45	8	3	94
A1	0.49	7	15	90
B1	0.61	14	21	89
B1.1	0.52	9	15	90
B1.2	0.26	4	3	95
B1.1 or B1.2	0.64	13	18	90
B3	0.69	7	8	96
C1	0.30	6	1	95
C2	0.61	2	1	99
C3	0.67	0	1	100

IRR between MG and AI for 409 observations. Average Cohen's kappa 0.52

	Cohen's kappa	% identifiedin the category Reviewer 1	Reviewer 2	Agreement
General				
Fracture	0.75	78	72	91
Malleolar	0.85	53	50	93
Tibia diaphyseal	0.50	7	17	89
Tibia distal	0.79	15	16	94
Fibula	0.53	9	16	90
Other bone	0.72	10	12	95
Malleolar				
A	0.54	9	7	93
B	0.71	33	38	87
C	0.48	10	8	91
A1	0.42	6	12	90
B1	0.67	17	19	90
B1.1	0.57	11	15	90
B1.2	0.05	6	4	91
B1.1 or B1.2	0.67	16	18	91
B3	0.44	9	9	91
C1	0.15	6	6	90
C2	0.17	3	13	87
C3	0.40	1	0	99

IRR between AS and AI for 409 observations. Average Cohen's kappa 0.45

	Cohen's kappa	% identifiedin the category Reviewer 1	Reviewer 2	Agreement
General				
Fracture	0.75	78	72	91
Malleolar	0.84	51	50	92
Tibia diaphyseal	0.25	3	17	86
Tibia distal	0.75	14	16	94
Fibula	0.55	9	16	90
Other bone	0.63	7	12	94
Malleolar				
A	0.41	5	7	93
B	0.70	35	38	86
C	0.34	11	8	89
A1	0.26	4	12	89
B1	0.57	11	19	89
B1.1	0.46	7	15	89
B1.2	0.27	4	4	94
B1.1 or B1.2	0.59	11	18	90
B3	0.38	6	9	91
C1	0.22	5	6	92
C2	0.18	5	13	86
C3	0.00	1	0	99

Other fracture classes

Fibular fracture. The outcome measures for the most important groups

	Cases n = 409	Sensitivity (%)	Specificity (%)	Youden's J	AUC (95% CI)
General	37	92	79	0.71	0.91 (0.85–0.96)
2	5	100	38	0.38	0.67 (0.45–0.90)
3	32	97	86	0.83	0.94 (0.92–0.97)
3A	16	94	78	0.72	0.90 (0.85–0.96)
3B	16	100	77	0.77	0.94 (0.90–0.98)
Weighted mean		94	79	0.73	0.90

Tibia diaphysis fractures. The outcome measures for the most important groups

	Cases n = 409	Sensitivity (%)	Specificity (%)	Youden's J	AUC (95% CI)
General	27	93	89	0.82	0.95 (0.92–0.98)
Base	16	94	84	0.77	0.94 (0.90–0.97)
1	16	100	87	0.87	0.94 (0.91–0.97)
Base	7	86	88	0.73	0.84 (0.61–0.97)
2	7	86	84	0.70	0.91 (0.82–0.97)
Weighted mean		93	87	0.80	0.93

Tibial distal fracture. The outcome measures for the most important groups

	Cases n = 409	Sensitivity (%)	Specificity (%)	Youden's J	AUC (95% CI)
General	65	79	90	0.69	0.90 (0.85–0.94)
Base	14	100	77	0.77	0.92 (0.88–0.96)
1	8	100	88	0.88	0.93 (0.90–0.96)
1.1	8	100	78	0.78	0.91 (0.85–0.96)
2	4	100	91	0.91	0.95 (0.92–0.98)
Base	8	100	59	0.59	0.87 (0.73–0.98)
1	8	100	64	0.64	0.87 (0.76–0.98)
1.1	6	100	63	0.63	0.85 (0.70–0.97)
1.2	2	100	95	0.95	0.97 (0.93–1.00)
Base	16	94	83	0.77	0.92 (0.87–0.96)
Weighted mean		90	83	0.72	0.90

Foot fractures. The outcome measures for the most important groups

	Cases n = 409	Sensitivity (%)	Specificity (%)	Youden's J	AUC (95% CI)
General	40	77	70	0.47	0.83 (0.75–0.89)
Calcaneus	17	76	94	0.70	0.90 (0.80–0.97)
Metatarsale	17	93	62	0.55	0.77 (0.70–0.85)
Metatars. V	14	91	66	0.57	0.78 (0.69–0.86)
Naviculare	3	100	86	0.86	0.92 (0.84–0.98)
Talus	4	100	76	0.76	0.90 (0.79–0.98)
Weighted mean		84	73	0.56	0.83
